# Extremely limited spatial and temporal utilization for wild Chinese alligator (*Alligator sinensis*)

**DOI:** 10.1098/rsbl.2025.0513

**Published:** 2025-11-12

**Authors:** Meng Li, Ke Sun, Ziyi Wang, Chongzhi Zhang, Yulin Gao, Song Zhang, GenJun Tu, Xiaobing Wu, Tao Pan

**Affiliations:** ^1^ College of Life Science, Anhui Normal University, Wuhu, People’s Republic of China; ^2^ The Anhui Provincial Key Laboratory of Biodiversity Conservation and Ecological Security in the Yangtze River Basin, Wuhu, People’s Republic of China; ^3^ Anhui Chinese Alligator National Nature Reserve, Xuancheng, People’s Republic of China; ^4^ National Long-term Scientific Research Base for Chinese Alligator Artificial Breeding and Protection in Anhui, Anhui Research Center for Chinese Alligator Reproduction, Xuancheng, People’s Republic of China

**Keywords:** Chinese alligator, nature reserve, suitability, movement rate, home range, spatial and temporal utilization

## Abstract

Habitat adaptation critically influences relocation success in endangered reptiles. Forestland is the dominant landscape in most of the nature reserves that are used for the relocation of the Chinese alligator. To evaluate habitat suitability and utilization, we analysed movement rates and home range of Chinese alligators within a core habitat of the nature reserve, concurrently assessing habitat suitability and carrying capacity. Results indicate severely restricted annual home ranges (average 7.415 ± 7.347 × 10^4^ m^2^ per alligator) and limited seasonal activity (March–October), with peak utilization confined to June–August in some individuals. Forestland cover significantly impeded the movement of Chinese alligators, and only 4.62% of the study area qualified as high-suitability habitat, supporting a carrying capacity of 147 individual Chinese alligators. Unsuitable habitat and climate drastically reduced the spatial and temporal utilization of the habitat. Conservation strategies should prioritize enhancing habitat quality and addressing the survival requirements and migration patterns of the Chinese alligator during the active period within nature reserves.

## 1. Introduction

The Chinese alligator (*Alligator sinensis*), an endemic Crocodilia species in China, is currently listed as critically endangered on the International Union for Conservation of Nature (IUCN) Red List. Its distribution is primarily confined to the lower reaches of the Yangtze River [1], where habitat degradation has intensified due to recent regional economic development. To protect the remaining wild population, the Chinese government established 433 km^2^ of nature reserves in 1982. Subsequent adjustments over four decades have resulted in seven reserves, each exceeding 200 km², located in Southern Anhui Province. Only one of these reserves is situated within an agricultural zone, the others are predominantly forested. Although forest-dominated landscapes reduce human–alligator conflict due to lower human influence [2], the scarcity of natural wetlands has necessitated the construction of artificial wetlands.

Successful reptile reintroduction hinges on habitat adaptation [3]. Currently, population recovery of the Chinese alligator relies on the captive-bred individuals released into nature reserves like Gaojingmiao (119.086° E, 31.021° N), Zhongqiao (118.382° E, 30.817° N) and Hongxing (118.727° E, 30.809° N) nature reserves [4,5]. Small parts of these nature reserves have been transformed into artificial conservation wetlands as core habitats for Chinese alligators.

Extinction vulnerability in reptiles arises from both habitat loss and life history constraints [6]. The slow-lived species exhibit reduced dispersal capabilities [7], and climate change and habitat fragmentation further threaten population recovery [8,9]. As a species with a slow life history [5], the Chinese alligator historically inhabited natural wetlands [10], but today most individuals are confined to artificial wetlands within nature reserves. Compared to natural wetlands, these areas are characterized by smaller ponds, fragmented patches of grassland and shrubland and surrounding forest, resulting in high habitat fragmentation. Studies of *Alligator mississippiensis* suggested that low habitat quality can reduce growth rates and that forested landscapes may limit population expansion [11,12]. Moreover, as a cold-blooded animal, the Chinese alligator is highly susceptible to extreme weather conditions [13]. We therefore hypothesized that habitat utilization of Chinese alligator is limited by both unsuitable habitat and climatic factors.

To test this hypothesis, we selected the core habitat in Gaojingmiao Nature Reserve as the study area, then used released Chinese alligator global positioning system (GPS) location data and dynamic Brownian bridge motion models (dBBMM) to analyse the movement rate and home range of the Chinese alligator. We also built a MaxEnt model for evaluating their suitability distribution. Finally, we estimated the carrying capacity of the study area based on species distribution records and habitat suitability modelling. The results provide insights for improving habitat construction and management strategies for the Chinese alligator.

## 2. Methods

### (a) Study area

**Figure 1.**
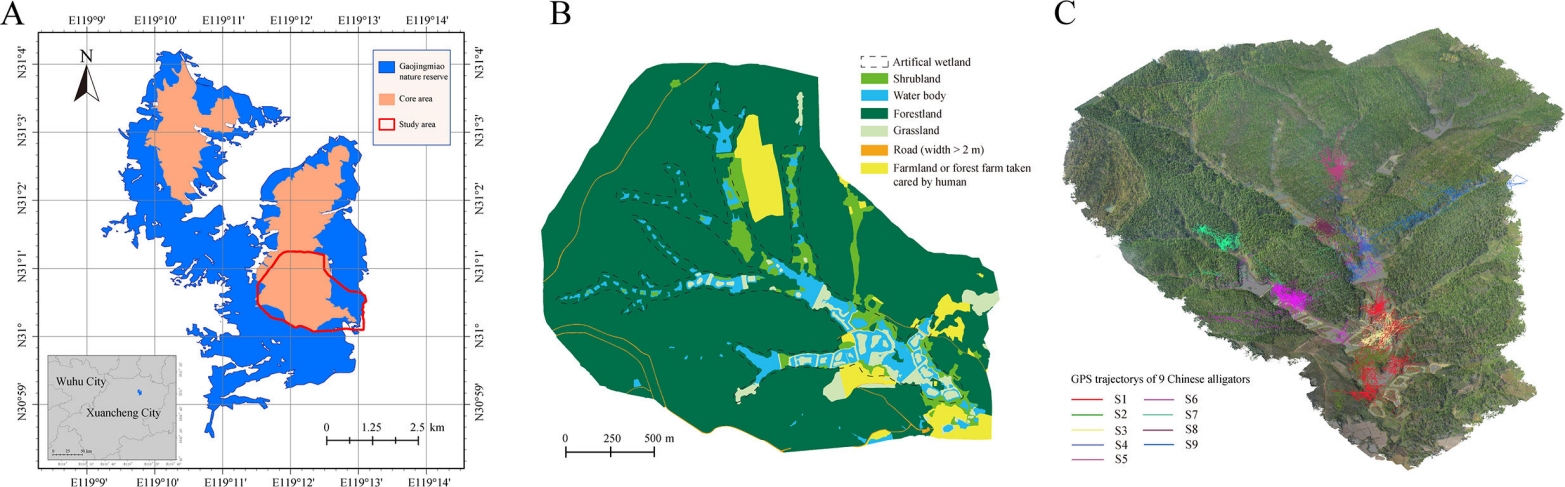
Study area and GPS locations for the Chinese alligators. (A) Study area—core habitat in the Gaojingmiao Nature Reserve. (B) Landscape of the study area. (C) Three-dimensional landscape model and GPS trajectories of the Chinese alligators.

The core habitat of Gaojingmiao Nature Reserve (119.086° E, 31.021° N) in Langxi County, Anhui Province, China, was selected as the study area (figure 1A). Encompassing 3.84 km² within the Gaojingmiao Forest Farm, the site averages 47 m elevation and is dominated by forest landscapes (80.2%). The other land cover types include water bodies (5.6%), farmland (5.5%), shrubland (4.5%), grassland (3.7%) and artificial wetlands were built along hilly valleys. Like most of the other nature reserves, Gaojingmiao Nature Reserve is a typical reserve area for Chinese alligators dominated by forest landscapes. According to data from the Chinese alligator survey in 2025, 93 adult Chinese alligators were recorded in the area.

### (b) Environmental variables

A high-resolution orthophoto (0.2 m GSD) was acquired in June 2023 using a DJI Phantom 4 RTK unmanned aerial vehicle. Then, according to the orthophoto and habitat survey, the landscape of the study area was artificially categorized using ArcGIS Pro v. 3.0 software (Environmental Systems Research Institute Inc., USA) into six types of patches: farmland, water body, forestland, grassland, road and shrubland (figure 1B). Next, the inside and outside distances to the edge of each landscape type were calculated using a Euclidean distance algorithm with a resolution of 0.2 m, and they were termed the farmland distance (DIFA), farmland depth (DPFA), water body distance (DIWA), distance from the shoreline inside the water body (DPWA), forestland distance (DIFO), forestland depth (DPFO), grassland distance (DIGR), grassland depth (DPGR), road distance (DIRO), road depth (DPRO), shrubland distance (DISH) and shrubland depth (DPSH). Correlation analysis confirmed that all pairwise correlations among environmental variables were below 0.7 (electronic supplementary material, figure S1), so all 12 variables were retained for modelling.

### (c) GPS tracking

During 2021–2022, Anhui Chinese Alligator National Nature Reserve released 32 GPS-tagged Chinese alligators into the core habitat of Gaojingmiao Nature Reserve (figure 1B). Custom-made HQZN GPS trackers (Global Messenger Inc., China) were attached to the tails of the alligators using two 2 cm wide leather belts (electronic supplementary material, figure S2). The devices measure 78 × 23.5 × 16.5 mm, weigh 46 g and have a location accuracy of 5 m. Due to environmental interference (e.g. signal obstruction) [14] and device damage, significant data loss occurred for some units. Therefore, nine Chinese alligators with relatively complete GPS data (>48 locations per month; duration >5 months) were selected for analysis. This cohort included three individuals (S1–S3) released in May 2021 and six (S4–S9) released in June 2022. Sex, tracking duration and total movement distance for each individual are provided in electronic supplementary material, table S1. GPS trajectories of these nine alligators (figure 1C) were resampled to a 1 day interval to standardize temporal resolution [15].

### (d) Movement rate and home range analysis

Movement rates and home range area are important parameters that quantify spatial utilization patterns and seasonal activity variations [16,17]. To assess the limitations of the spatial and temporal utilization of the Chinese alligator, daily movement rates of the nine Chinese alligators were derived from GPS trajectories resampled at 1 day intervals using MATLAB 2021 (MathWorks Inc., USA). Monthly and total home ranges were estimated at the 90% confidence level using the dBBMM method implemented in the R package ‘move’ (https://bartk.gitlab.io/move). Unlike kernel density methods, dBBMM is based on conditional random walk theory between successive positions, offering improved accuracy for trajectory data [18].

### (e) Climate impact assessment

Climate significantly influences the behaviour of the Chinese alligator. To investigate its impact on the temporal utilization patterns of Chinese alligators, this study obtained the average monthly temperature and precipitation data of the lower reaches of the Yangtze River between 2021 and 2023 from the China Meteorological Data Network (http://data.cma.cn; weather station number CHM00058338, 31.1499° N, 118.5799° E). Using PASW Statistics v. 18.0 (IBM Inc., USA), partial correlation analysis was conducted between temperature and movement rate. This analysis aimed to isolate the influence of climate factors on the movement patterns of Chinese alligator.

### (f) Habitat suitability evaluation

To comprehensively evaluate habitat suitability for Chinese alligators in the study area, the MaxEnt model was employed. This model is known for its high predictive accuracy, particularly when the number of species locations are limited [9]. Based on 12 environmental variable layers and the sites of adult alligators from the field survey, habitat suitability was assessed using MaxEnt v. 3.4.4 software from the Java platform. The model was configured with a maximum of 2000 iterations and 10 000 background points. The prediction effect was evaluated using a cross-validation method with five replicates, and the ‘Cloglog’ parameter was used as the model output for habitat suitability. The model’s accuracy was assessed using area under the receiver operating characteristic curve (AUC) values. Areas with a habitat suitability greater than 0.6 were classified as high-suitability areas [19]. The proportion of high-suitability areas within the total home ranges of the nine tracked alligators was calculated to evaluate their influence on alligator activity.

### (g) Carrying capacity evaluation

Carrying capacity refers to the stable population size supported by a given habitat [20]. It can be influenced by the balance between birth rates and death rates, as well as the balance between migration in and migration out. Annual surveys indicate a relatively stable alligator population in the study area, with equilibrium between reintroduction and dispersal. Due to the difficulty in spotting all the alligators, carrying capacity was estimated via habitat suitability modelling (21). The 2025 survey provided precise coordinates for 93 adult alligators, which were used in this analysis.

A random forest (RF) model was selected for evaluating the habitat carrying capacity of Chinese alligators in the study area due to its high accuracy and wide applicability [22]. This model is particularly effective when there are obvious nonlinearities and interactions between variables [23], and can regress the continuous strain variables. Based on the 93 alligator locations, the probability density distribution of Chinese alligators was calculated using the kernel density analysis algorithm in ArcGIS Pro v. 3.0 software. Within the study area, 2000 random points were generated, and their environmental parameters and probability densities were extracted. An RF model was then established using Matlab v. 2021 software, with parameter optimization ranges of 1–100 for the number of trees and 0.1–5.0 for the minimum leaf size. Prediction effect of the model was evaluated using a cross-verification method with five replicates, and its accuracy was assessed using the *R*^2^ value. A predicted probability density distribution map with a resolution of 0.2 m was finally produced by the established model. The habitat carrying capacity was subsequently predicted through the calculation of the cumulative sum of all pixel values within the map.

## 3. Results

### (a) Movement rate and home range

**Figure 2.**
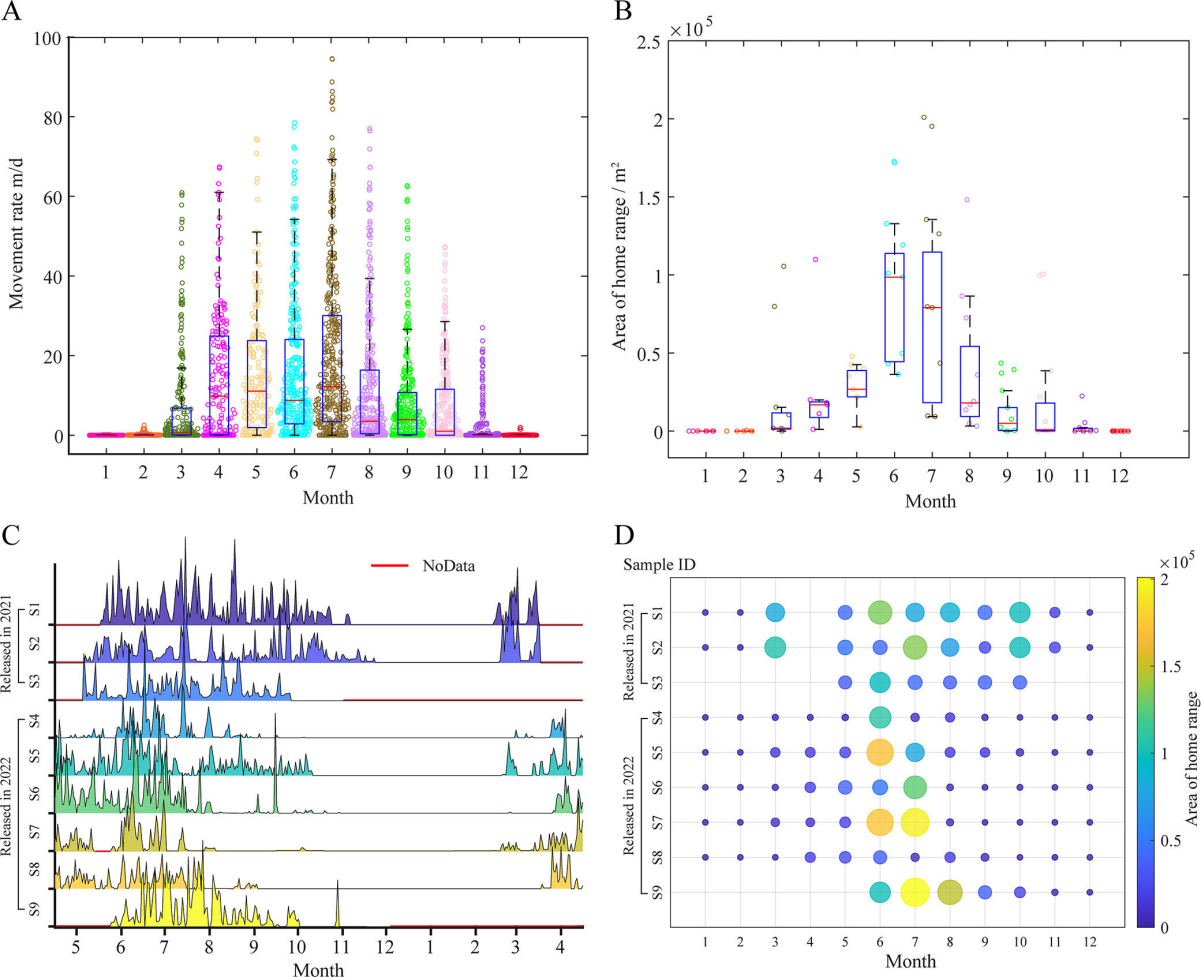
Movement rate and home range area of Chinese alligators in the study area. (A) Movement rate in each month. (B) Area of home range in each month. (C) Movement rate change for each Chinese alligator. (D) Area of home range change for each Chinese alligator.

The movement rate and home range serve as key indicators of Chinese alligator activity levels. Figure 2 illustrates the annual variations in both movement rate and home range. As depicted in figure 2A,C, the mean movement rate demonstrated pronounced seasonal fluctuations, with elevated values observed from May through August and reduced activity from September to April of the following year. Notably, substantial inter-individual variation was evident in movement patterns. Some Chinese alligators (S4, S7 and S8) exhibited dramatic declines in movement rate following July, while all individuals except S1 and S2 showed near-zero movement rates after October.

The average total home range area estimated by dBBMM at 90% confidence level was 7.415 ± 7.347 × 10^4^ m^2^. Figure 2B,D reveal distinct temporal patterns: individuals S1 and S2 (released in 2021) maintained relatively consistent home ranges from March to October, whereas those released in 2022 concentrated their activity primarily between June and August. This temporal discrepancy may reflect interannual precipitation differences. Both S4 and S8 maintained exceptionally small home ranges throughout the observation period, potentially attributable to individual health conditions or limited habitat suitability.

### (b) Climatic influences on movement dynamics

The electronic supplementary material, figure S3, presents comparative data on monthly temperature, precipitation and mean movement rates for alligators released in 2021 versus 2022. Although temperature patterns were similar between years, precipitation during the 2022 rainy season (July–August) was lower. Partial correlation analysis showed a strong positive relationship (*R* = 0.851, *p* < 0.001) between temperature and movement rate when controlling for precipitation. Reduced rainfall during the 2022 rainy season (<100 mm month^−1^) correlated with decreased post-rainy season movement, suggesting that dry conditions may constrain alligator activity.

### (c) Habitat suitability and carrying capacity

**Figure 3.**
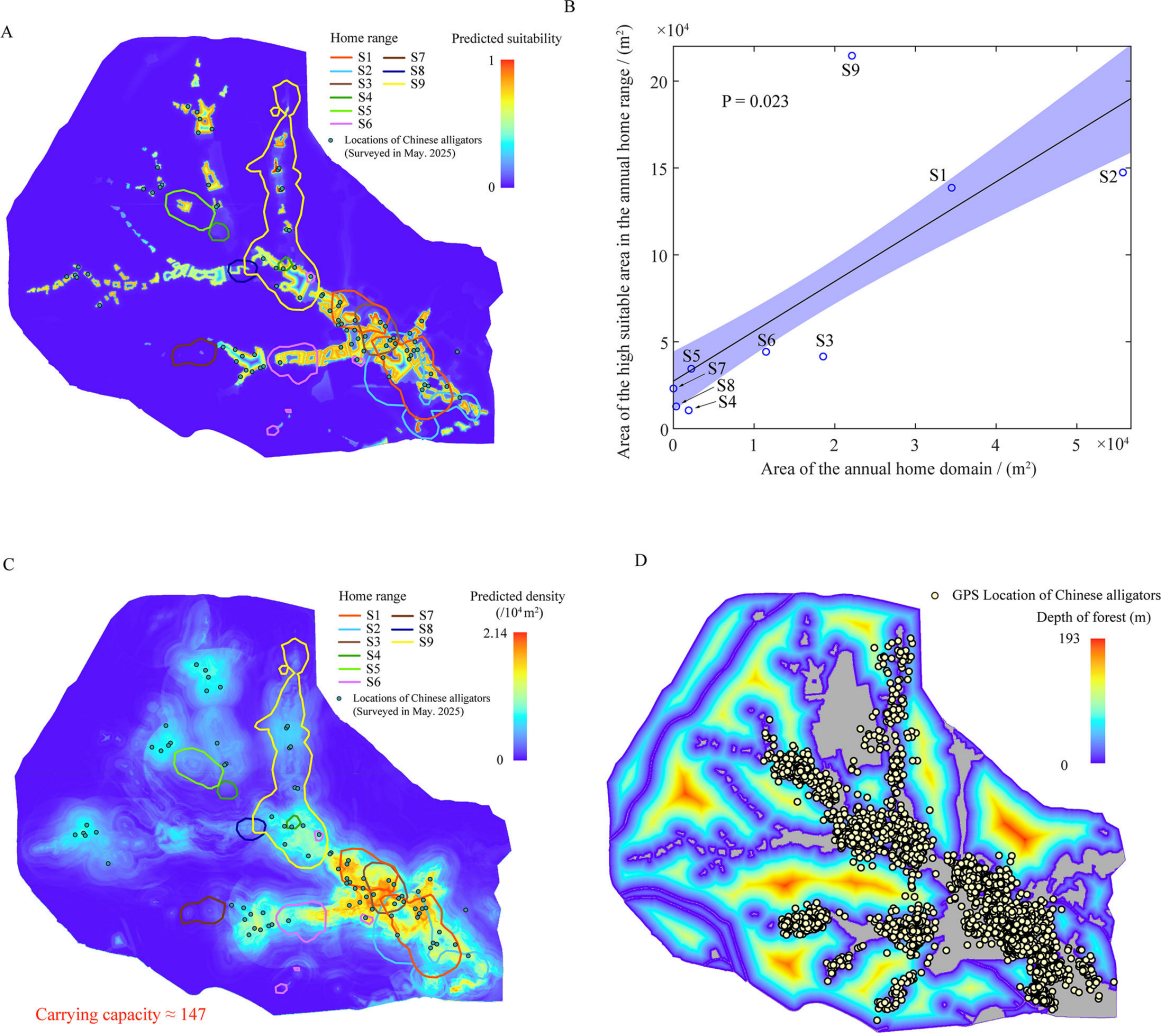
Suitability and carrying capacity of the study area. (A) Suitability distribution. (B) Relationship between home range and high suitability habitat area. (C) Capacity distribution of Chinese alligator. (D) Barrier effect of forestland on the Chinese alligator movement. (A,C) Circles of different colours represent the total home range of each Chinese alligator calculated using the dynamic Brownian bridge model at 90% confidence level.

The MaxEnt model predicted habitat suitability with high accuracy (AUC = 0.975 ± 0.005). Suitable areas were heterogeneous, with high suitability along water bodies and low suitability in forestland and farmland (figure 3A). High-suitability areas (defined by above 0.6 threshold) constituted only 4.62% of the total area. Figure 3B demonstrates a positive correlation between total home range area and the proportion of high-suitability habitat contained within it. Insufficient availability of optimal habitat likely drives the constrained spatial and temporal utilization patterns of reintroduced Chinese alligators.

Carrying capacity distribution modelled by RF (figure 3C) exhibits spatial congruence with MaxEnt-derived suitability patterns. The predicted carrying capacity for adult alligators is 147 individuals (current population: 93), indicating limited expansion potential. Notably, alligator dispersal into forested areas was restricted to marginal zones, with an average penetration depth of 14.2 m (only depth of forest exceeding 1 m was counted) (figure 3D). This confirms that forest landscapes function as effective dispersal barriers.

## 4. Discussion

This study systematically evaluated the temporal–spatial utilization and suitability of habitat for Chinese alligators in nature reserve. The observed home range area (7.415 ± 7.347 × 10⁴ m^2^) is notably smaller than that of most other Crocodilia, such as *Alligator mississippiensis* (>30.0 × 10^4^ m^2^) [24–26], *Caiman latirostris* (43.2 ± 78.6 × 10^4^ m^2^) [27], *Mecistops leptorhynchus* (median: 17.91 × 10^4^ m^2^) [28] and female *Crocodylus acutus* (8.44 ± 3.23 × 10^4^ m^2^) [16]. The active period of Chinese alligators was restricted to March–October, with some individuals showing peak spatial utilization only during June–August. The estimated carrying capacity of 147 individuals suggests severe ecological constraints. These limitations appear primarily attributable to unsuitable habitat conditions and climate.

### (a) Unsuitable habitat

The nature reserves for Chinese alligator were established in 1982, and now have a total area of more than 200 km^2^. However, currently, the Chinese alligator’s home range in the nature reserve has no obvious improvement compared to the home range detected in 2003 [5], indicating the limited suitability improvement of the core habitat. Excluding Changle Nature Reserve, the remaining six nature reserves are predominantly forestland (electronic supplementary material, figure S4). This forestland dominance has resulted in narrowly elongated high-suitability habitat zones within core areas. Smooth ecological corridors and accessible water bodies can improve food resources and population exchange capacity [8]. The slow movement speed of Chinese alligators hinders their ability to find food and mates [5], and a high canopy density may make it more difficult to thermoregulate using sunlight [29]. These constraints severely limit carrying capacity in core habitats and undermine wild population restoration through reintroduction programmes.

### (b) Climate

Temperature constitutes the primary climatic factor governing the Chinese alligator behavioural rhythms. Compared to other *Crocodylus* species, the Chinese alligator inhabits the highest latitudes and undergoes obligatory hibernation from November to April [30]. As shown in electronic supplementary material, table S1, Chinese alligators experience the lowest average habitat temperatures among *Crocodylus* species, and low temperature is the main reason for their limited temporal utilization [31]. Moreover, the species inhabits a subtropical monsoon climate characterized by the unique Meiyu rainy season during summer [32]. Inadequate rainfall during this period may lower pond water levels and reduce alligator activity. These factors collectively result in a significantly restricted wild active period relative to biological requirements. Consequently, Chinese alligators may have only 3–4 months for reproduction and energy accumulation before winter. In degraded habitats, insufficient food intake leads to stunted growth, overwinter survival challenges and population decline.

### (c) Implications for habitat management

Improving the spatial and temporal utilization of habitats requires expanding water bodies, restoring grassland and shrubland and establishing ecological corridors [33]. These measures would enhance feeding, breeding and hibernation habitats. Ensuring adequate food and water during low-rainfall summers is critical. Conservation strategies must address both the immediate needs of released populations and long-term habitat restoration. Protected areas and habitat management are vital for Crocodilia conservation globally, as seen in the Everglades National Park for *C. acutus* [34] and the Katerniaghat Wildlife Sanctuary for *Gavialis gangeticus* [35]. Species such as *Crocodylus palustris* in India and *Caiman* species in Bolivia have benefitted from effective habitat management [36,37]. However, climate change and deforestation threaten these gains, as projected for *Caiman yacare* [38]. Continued efforts in habitat restoration and management are essential for Crocodilia conservation worldwide.

## 5. Limitation

Due to the technical limitations of GPS trackers, it is currently impossible to obtain complete GPS data for the Chinese alligator, which makes it difficult to analyse their behaviour in their habitats. In the future, the GPS tracker should be improved to make it more suitable for tracking Chinese alligator individuals and ensure continuous data return.

## 6. Conclusion

In conclusion, the habitat suitability of the Chinese alligator is limited in the nature reserves, and the unsuitable habitat affects the spatial utilization of the habitat. Climate factors such as temperature and precipitation affect the behavioural rhythm of Chinese alligators and further affect the temporal utilization of the habitat. Currently, the nature reserve has a low carrying capacity of Chinese alligators, and the carrying capacity is estimated to be only 147 individuals in the study area, with an area of 3.84 km^2^. We suggest that the protection of wild Chinese alligator populations should focus on range expansion and improvement of habitat quality, and the management of wild Chinese alligators should pay attention to survival needs and population trends during their active periods.

## Data Availability

Environmental variable layers, the GPS data of nine Chinese alligators and the Matlab Code used in this study have been deposited in a Figshare dataset [39]. Supplementary material is available online [40].
